# Predictive Value of DXA Appendicular Lean Mass for Incident Fractures, Falls, and Mortality, Independent of Prior Falls, FRAX, and BMD: Findings from the Women's Health Initiative (WHI)

**DOI:** 10.1002/jbmr.4239

**Published:** 2021-01-28

**Authors:** Nicholas C Harvey, John A Kanis, Enwu Liu, Cyrus Cooper, Mattias Lorentzon, Jennifer W Bea, Laura Carbone, Elizabeth M Cespedes Feliciano, Deepika R Laddu, Peter F Schnatz, Aladdin H Shadyab, Marcia L Stefanick, Jean Wactawski‐Wende, Carolyn J Crandall, Helena Johansson, Eugene McCloskey

**Affiliations:** ^1^ MRC Lifecourse Epidemiology Unit University of Southampton Southampton UK; ^2^ NIHR Southampton Biomedical Research Centre University of Southampton and University Hospital Southampton NHS Foundation Trust Southampton UK; ^3^ Centre for Metabolic Bone Diseases University of Sheffield Sheffield UK; ^4^ Mary McKillop Institute for Health Research Australian Catholic University Melbourne Australia; ^5^ NIHR Biomedical Research Centre University of Oxford Oxford UK; ^6^ Geriatric Medicine, Department of Internal Medicine and Clinical Nutrition Institute of Medicine, University of Gothenburg Gothenburg Sweden; ^7^ Geriatric Medicine Sahlgrenska University Hospital Mölndal Sweden; ^8^ University of Arizona Cancer Center Tucson AZ USA; ^9^ Department of Medicine, Division of Rheumatology, J Harold Harrison, MD, Distinguished University Chair in Rheumatology Medical College of Georgia at Augusta University Augusta GA USA; ^10^ Division of Research Kaiser Permanente Northern California Oakland CA USA; ^11^ Department of Physical Therapy College of Applied Health Sciences, University of Illinois at Chicago Chicago IL USA; ^12^ Reading Hospital / Tower Health Reading PA USA; ^13^ University of California San Diego CA USA; ^14^ Department of Medicine (Stanford Prevention Research Center) Stanford School of Medicine, Stanford University Stanford CA USA; ^15^ University at Buffalo, the State University of New York Buffalo NY USA; ^16^ David Geffen School of Medicine at the University of California Los Angeles CA USA; ^17^ Centre for Integrated Research in Musculoskeletal Ageing (CIMA) Mellanby Centre for Bone Research, University of Sheffield Sheffield UK

**Keywords:** APPENDICULAR LEAN MASS, BMD, DXA, FRACTURE, FRAX, OSTEOPOROSIS: EPIDEMIOLOGY

## Abstract

In the Women's Health Initiative (WHI), we investigated associations between baseline dual‐energy X‐ray absorptiometry (DXA) appendicular lean mass (ALM) and risk of incident fractures, falls, and mortality (separately for each outcome) among older postmenopausal women, accounting for bone mineral density (BMD), prior falls, and Fracture Risk Assessment Tool (FRAX^®^) probability. The WHI is a prospective study of postmenopausal women undertaken at 40 US sites. We used an extension of Poisson regression to investigate the relationship between baseline ALM (corrected for height^2^) and incident fracture outcomes, presented here for major osteoporotic fracture (MOF: hip, clinical vertebral, forearm, or proximal humerus), falls, and death. Associations were adjusted for age, time since baseline and randomization group, or additionally for femoral neck (FN) BMD, prior falls, or FRAX probability (MOF without BMD) and are reported as gradient of risk (GR: hazard ratio for first incident fracture per SD increment) in ALM/height^2^ (GR). Data were available for 11,187 women (mean [SD] age 63.3 [7.4] years). In the base models (adjusted for age, follow‐up time, and randomization group), greater ALM/height^2^ was associated with lower risk of incident MOF (GR = 0.88; 95% confidence interval [CI] 0.83–0.94). The association was independent of prior falls but was attenuated by FRAX probability. Adjustment for FN BMD *T*‐score led to attenuation and inversion of the risk relationship (GR = 1.06; 95% CI 0.98–1.14). There were no associations between ALM/height^2^ and incident falls. However, there was a 7% to 15% increase in risk of death during follow‐up for each SD greater ALM/height^2^, depending on specific adjustment. In WHI, and consistent with our findings in older men (Osteoporotic Fractures in Men [MrOS] study cohorts), the predictive value of DXA‐ALM for future clinical fracture is attenuated (and potentially inverted) after adjustment for femoral neck BMD *T*‐score. However, intriguing positive, but modest, associations between ALM/height^2^ and mortality remain robust. © 2021 The Authors. *Journal of Bone and Mineral Research* published by Wiley Periodicals LLC on behalf of American Society for Bone and Mineral Research (ASBMR).

## Introduction

Dual‐energy X‐ray absorptiometry (DXA)–derived appendicular lean mass (ALM) is central to the more than 10 current operational definitions of sarcopenia.^(^ ^1^, ^2^ ^)^ Concerns over the predictive value of DXA ALM for incident health outcomes such as fractures, falls, and death have led to more recent sarcopenia definitions incorporating measures of physical performance/function and muscle strength, rather than being based solely on ALM.^(^ ^1^, ^2^ ^)^ Indeed, the most recent European working group consensus definition focuses principally on physical function as the initial criterion for sarcopenia definition^(^ ^3^ ^)^ and the 2020 US Sarcopenia Definitions and Outcomes Consortium approach dispenses with ALM entirely.^(^ ^4^, ^5^ ^)^ There is evidence that DXA ALM is variably predictive of fracture outcomes in men, particularly when femoral neck bone mineral density (BMD) is also included in the analyses.^(^ ^1^ ^)^ For example, we have demonstrated recently in the US, Sweden, and Hong Kong Osteoporotic Fractures in Men (MrOS) study cohorts that DXA ALM, both as a crude measure and normalized for height, is only modestly predictive of incident fractures; when femoral neck (FN) BMD *T*‐score is also considered, the predictive value is attenuated to the null, or even, in the case of hip fracture, inverted, such that greater DXA ALM becomes a risk factor for fracture.^(^ ^6^ ^)^ Similar findings have emerged from the US Health ABC cohort,^(^ ^7^ ^)^ with supportive evidence in women from Women's Health Initiative (WHI)^(^ ^8^ ^)^ and Framingham,^(^ ^9^ ^)^ but whether these considerations apply to the related outcomes of incident falls and mortality is unclear. Additionally, the independent value of ALM in predicting fracture outcomes, after controlling for falls, Fracture Risk Assessment Tool (FRAX^®^) probability, or femoral neck BMD has to date not been quantified in women. Given that the acquisition of DXA ALM requires an additional scan, which may take between 5 and 15 minutes depending on the instrument and the size of the participant, if it does not add useful risk information for a particular outcome over and above more easily obtainable measures, such as femoral neck BMD (for which the scanning time is usually less than 30 seconds), FRAX probability, or history of falls, then its value as part of sarcopenia definitions is questionable, at least in the context of that outcome.^(^ ^1^ ^)^ Building on our previous findings in men, the aim of the present study was to examine, in a large population of older women, first whether DXA ALM is predictive of incident fractures independent of current measures such as femoral neck BMD, prior falls, and FRAX probability, and second to elucidate associations between baseline DXA ALM and incident falls and mortality.

## Materials and Methods

### Participants

The WHI is a prospective health study in the United States undertaken at 40 centers and focused on strategies for preventing heart disease, breast and colorectal cancer, and osteoporotic fractures in postmenopausal women. The WHI included 161,808 women aged 50 to 79 years at baseline, who were postmenopausal and with predicted survival of 3 or more years. The WHI structure and methods have been presented in detail previously.^(^ ^8^, ^10^, ^11^ ^)^ In brief, women were enrolled at 40 US clinical centers into one or more randomized clinical trials (low‐fat diets [DM], hormone therapy [HT], or calcium and vitamin D [CAD] supplementation). Women who were ineligible or not interested in participating in the clinical trials were enrolled in the observational study (OS). In this analysis, we studied the cohort of women who had undergone DXA assessment at baseline, at one of three centers, spanning participants from DM, HT, CAD, and OS studies, described below. The analysis dataset comprised all individuals from the four WHI studies for whom required exposure and outcome data were available. No other inclusion/exclusion criteria were applied.

### Exposure variables

At baseline, height, using a wall‐mounted stadiometer (to the nearest 0.1 cm), and weight, using a balance beam scale (to the nearest 0.1 kg), were measured, and body mass index (BMI) was calculated as kg/m^2^. Hip and waist circumferences were measured to the nearest 0.5 cm, the latter at the level of the umbilicus over nonbinding undergarments. The WHI questionnaire was administered at baseline to collect information about current smoking, number and type of medications, fracture history, family history of hip fracture, past medical history (rheumatoid arthritis), and high consumption of alcohol (three or more glasses of alcohol‐containing drinks per day). Previous fracture at baseline was documented as all fractures occurring after the age of 55 years. Glucocorticoid exposure was recorded as use at least three times per week in the month preceding the baseline assessment. Given their rarity in this cohort, apart from glucocorticoid use and rheumatoid arthritis (both FRAX input variables), we did not consider secondary causes of osteoporosis and the “secondary osteoporosis” input variable for FRAX probability calculation was set to no for all women.^(^ ^6^ ^)^ The number of falls during the 12 months preceding the baseline visit was recorded by self‐assessment questionnaire (past falls). In the WHI Bone Density Study, BMD and body composition, including ALM, were measured at three US clinic sites (Pittsburgh, PA; Birmingham, AL; Phoenix/Tucson, AZ), using Hologic QDR 2000, 2000+ or 4500 instruments (Hologic, Bedford, MA, USA), in participants of all three component trials and the observational study. A standardized procedure for participant positioning and scan analysis was used at all centers. Phantom scans, scans with specific problems, and a random sampling of scans were reviewed in the WHI quality assurance program to monitor machine and technician, and cross‐calibration was undertaken.^(^ ^8^, ^10^, ^11^ ^)^ The DXA assessment also generated total body fat mass (kg). In the analysis cohort, 10‐year probability of fracture (FRAX major osteoporotic fracture [hip, humerus, clinical vertebral, or forearm]) was calculated using clinical risk factors described above with and without femoral neck BMD entered into the US‐specific FRAX model.

### Fracture, fall, and death outcomes

Fractures were initially reported by participants and subsequently verified by radiology review or operative reports by centrally trained and blinded physician adjudicators at each clinical center.^(^ ^11^, ^12^ ^)^ Final adjudication of hip fractures was performed centrally by blinded adjudicators. Incident falls were assessed by questionnaire at follow‐up (at least annually), using the question, “Since your last medical update, how many times did you fall and land on the floor or ground?” (with options ranging from 0 to 3 or more). Deaths were ascertained from registry data and reports from family members/physicians.

### Statistical methods

Clinical outcomes comprised: any fracture, osteoporotic fracture (OF: defined consistent with Kanis et al. 2001^(^ ^13^ ^)^ as clinical vertebral, pelvis, humerus, sacrum/coccyx, scapula, sternum, hip, other femoral fractures, tibia, fibula, distal forearm), major osteoporotic fracture (MOF: hip, clinical vertebral, humerus or forearm) and hip fracture, incident falls, and death. An extension of Poisson regression models^(^ ^14^ ^)^ was used to study the association between the ALM/height^2^, FRAX, prior falls, BMD, and the risk of incident outcomes. ALM/height^2^ was first standardized (in the whole analysis cohort) to a normally distributed variable with mean 0.00 and SD 1.00. All associations were adjusted for current age, current time since baseline, randomization (to low‐fat diet, hormone therapy, calcium and vitamin D supplementation, or placebo), and participation in the observational study. In contrast to logistic regression, the Poisson regression uses the length of each individual's follow‐up period and the hazard function is assumed to be exp(β_0_ + β_1_ · current time from baseline + β_2_ · current age + β_3_ · variable of interest). The observation period of each participant was divided into intervals of 1 month. One fracture per person and time to the first fracture were counted, and time at risk was censored at the time of first fracture, loss to follow‐up, death, or end of follow‐up. Unlike a Cox model, the Poisson model uses a data duplication method, accounting for the competing mortality risk for fracture risk prediction.^(^ ^15^ ^)^

We initially investigated the predictive value of ALM/height^2^ adjusted only for current age and follow‐up time. Subsequently, we used multivariate models to investigate the predictive value of ALM/height^2^ independent of FRAX, prior falls, or BMD. Interactions between ALM/height^2^ and current age and between ALM/height^2^ and current time since baseline were also investigated in order to elucidate whether the associations between ALM/height^2^ and outcomes differed by age or time since baseline. DXA total fat mass and waist/hip ratio were used in post hoc exploratory models with mortality as the outcome. These exploratory analyses were undertaken to further investigate emergent findings and therefore were not documented in the original analysis plan.

The associations between ALM and outcomes are presented as a gradient of risk (GR = hazard ratio per SD) together with 95% confidence intervals (CI). Two‐sided *p* values were used for all analyses and *p* < .05 was considered to be statistically significant.

## Results

### Characteristics of the participants

We studied 11,187 women (Table 1). Their mean age was 63.3 years (SD 7.4 years) and 17% had experienced a prior fracture since the age of 55 years. In the preceding 12 months, 33% had experienced a fall. Average follow‐up time was 14.1 years, with a maximum of 21.5 years. Supplemental Table SS1 documents the baseline characteristics by quarter of ALM/height^2^.

**Table 1:** Baseline Characteristics of the Participants

	No. with data	Mean/*n*	SD/%	Range
Age (years)	11,187	63.3	7.4	50–79
Height (cm)	11,187	161.6	6.4	98.5–212.0
BMI (kg/m^2)^	11,180	28.2	5.9	14.3–69.1
Prior fracture	7685	1325	17%	
Parental history of hip fracture	10,927	1326	12%	
Current smoking	11,029	889	8%	
Corticosteroids	11,187	98	1%	
Rheumatoid arthritis	10,384	607	6%	
Excess alcohol intake	11,151	324	3%	
Femoral neck BMD (g/cm^2^)	11,187	0.72	0.13	0.3–1.5
FRAX MOF without BMD	11,187	9.8	6.9	0.7–66.9
FRAX MOF with BMD	11,186	10.4	7.7	1.0–79.3
Prior falls	10,067	3307	33%	
ALM (g)	11,187	14,769	2809	7742–31,903
ALM/height^2^ (g/cm^2^)	11,187	0.56	0.10	0.3–1.7
ALM/height^2^ – normalized	11,187	0.00	1.00	−3.4–6.7
*During follow‐up*				
Length of follow‐up	11,187	14.1	5.6	0.0–21.5
Any fracture	11,187	1692	15%	
Osteoporotic fracture	11,187	1225	11%	
MOF	11,187	1024	9%	
Hip fracture	11,187	344	3%	
Falls	11,144	7720	69%	
Death	11,187	2236	20%	

BMI = body mass index; BMD = bone mineral density; FRAX = Fracture Risk Assessment Tool; MOF = major osteoporotic fracture; ALM = appendicular lean mass.

### Associations between DXA ALM and incident fracture

Associations between ALM/height^2^ and risk of incident fractures are presented in Table 2. Greater ALM/height^2^ was associated with lower risk of incident fracture, whether this was categorized as any clinical fracture, osteoporotic fracture, MOF, or hip fracture. The hazard ratio per standard deviation increase (GR) was similar for all fracture types with the greatest magnitude of association for hip fracture (GR = 0.81 [95% CI 0.71–0.91]), and weakest association for any clinical fracture (GR = 0.91 [95% CI 0.87–0.96]).

**Table 2:** Associations Between DXA ALM/Height^2^ and Incident Fracture Outcomes

Exposure (SD)	Adjustment	Any fx	Ost fx	MOF fx	Hip fx
ALM/height^2^	Age, FU time	**0.91 (0.87, 0.96)** ***p* < .001**	**0.90 (0.85, 0.96)** ***p* < .001**	**0.88 (0.83, 0.94) *p* < .001**	**0.81 (0.71, 0.91)** ***p* < .001**
	+ prior falls	**0.93 (0.88, 0.98)** ***p* = .0049**	**0.92 (0.87, 0.98)** ***p* = .015**	**0.89 (0.83, 0.96) *p* = .0013**	**0.81 (0.72, 0.92)** ***p* = .0010**
	or + FRAX without BMD	0.97 (0.92, 1.02) *p* = .23	0.97 (0.91, 1.03) *p* = .29	0.95 (0.89, 1.02) *p* = .15	**0.86 (0.76, 0.98)** ***p* = .019**
	or + FRAX with BMD	0.98 (0.93, 1.03) *p* > .30	0.98 (0.92, 1.04) *p* > .30	0.96 (0.90, 1.03) *p* = .25	**0.88 (0.78, 1.00)** ***p* = .044**
	or + FN BMD	1.05 (1.00, 1.11) *p* = .064	**1.07 (1.01, 1.15)** ***p* = .032**	1.06 (0.98, 1.14) *p* = .12	1.00 (0.88, 1.14) *p* > .30

DXA = dual‐energy X‐ray absorptiometry; ALM = appendicular lean mass; fx = fracture; Ost = osteoporotic; MOF = major osteoporotic fracture; FU = follow‐up; FRAX = Fracture Risk Assessment Tool; BMD = bone mineral density; FN = femoral neck.

Models are presented adjusted for age and follow‐up time alone and then additionally for either prior falls, FRAX MOF probability without BMD, FRAX MOF probability with BMD, or femoral neck BMD *T*‐score. Associations where *p* < 0.05 are in bold. Data are gradient of risk (hazard ratio per SD) and 95% confidence interval.

### Fracture outcomes and adjustment for prior falls, FRAX probability, or femoral neck BMD *T*‐score

**Fig 1:**
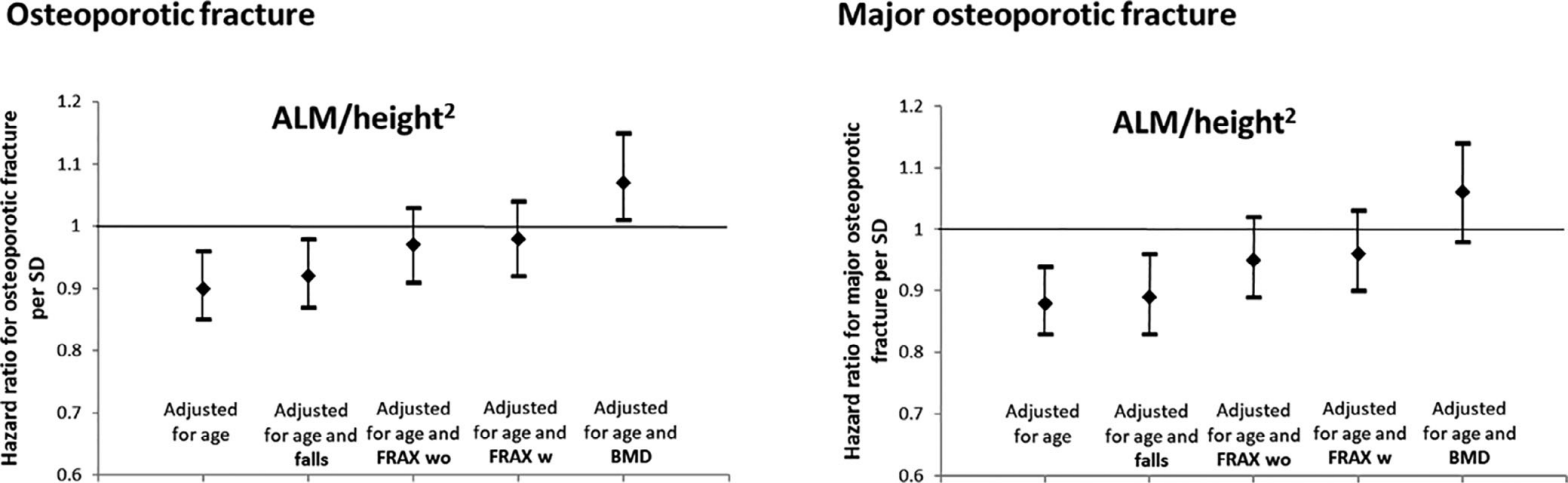
Associations between DXA appendicular lean mass (ALM)/height^2^ (SD) and incident fracture outcomes. Models are presented adjusted for age and follow‐up time alone and then additionally for either prior falls, Fracture Risk Assessment Tool (FRAX) major osteoporotic fracture (MOF) probability without bone mineral density (BMD), FRAX MOF probability with BMD, or femoral neck BMD *T*‐score. Data are gradient of risk (hazard ratio per SD) and 95% confidence interval.

Associations between ALM/height^2^ and incident fracture were not materially changed by inclusion of prior falls in the regression models (Table 2). Adjustment for FRAX probability of major osteoporotic fracture calculated with or without femoral neck BMD attenuated the associations to close to unity except for hip fracture (GR = 0.88 [95% CI 0.78–1.00] and GR = 0.86 [95% CI 0.76–0.98]) for FRAX with and without BMD, respectively). Adjustment for femoral neck BMD *T*‐score led to the point estimates for the GR becoming greater than unity, which were statistically significant for all fracture outcomes other than hip fracture. Fig. 1 documents these associations for the outcomes of osteoporotic fracture and major osteoporotic fracture.

### Associations between DXA ALM and incident falls

The relationships between ALM/height^2^ and incident falls are summarized in Table 3. There were no statistically significant associations.

**Table 3:** Associations Between DXA ALM/height^2^ and Incident Falls and Death

Exposure (SD)	Adjustment	Falls	Death
ALM/height^2^	Age, FU time	0.98 (0.96, 1.01) *p* = .18	**1.13 (1.08, 1.18)** ***p* < .001**
	+ prior falls	0.98 (0.96, 1.01) *p* = .15	**1.12 (1.07, 1.17)** ***p* < .001**
	or + FRAX without BMD	1.00 (0.98, 1.03) *p* > .30	**1.14 (1.09, 1.20)** ***p* < .001**
	or + FRAX with BMD	1.00 (0.98, 1.03) *p* > .30	**1.15 (1.10, 1.20)** ***p* < .001**
	or + FN BMD	0.99 (0.97, 1.02) *p* > .30	**1.13 (1.08, 1.19)** ***p* < .001**

DXA = dual‐energy X‐ray absorptiometry; ALM = appendicular lean mass; FU = follow‐up; FRAX = Fracture Risk Assessment Tool; BMD = bone mineral density; FN = femoral neck.

Models are presented adjusted for age and follow‐up time alone and then additionally for either prior falls, FRAX MOF probability without BMD, FRAX MOF probability with BMD, or femoral neck BMD *T*‐score. Associations where *p* < 0.05 are in bold. Data are gradient of risk (hazard ratio per SD) and 95% confidence interval.

### Associations between DXA ALM and incident mortality

In contrast, for each standard deviation greater ALM/height^2^, the risk of death during follow‐up was 13% higher (GR = 1.13 [95% CI 1.08–1.18]; Table 3). This was not materially changed by adjustment for either prior falls, FRAX, or femoral neck BMD. We investigated whether this positive association between ALM/height^2^ and death might be explained by DXA total fat mass or measured waist/hip ratio, but additional adjustment (with age and follow‐up time) for these variables did not materially alter the relationship (total fat mass: GR = 1.10 [95% CI 1.05–1.16); waist/hip ratio GR = 1.07 [95% CI 1.02–1,12]). Furthermore, findings were consistent with ALM rather than ALM/height^2^ as the exposure.

### Interactions with age and follow‐up time

We observed no evidence that the relationship between DXA ALM/height^2^ and outcomes varied by age except for hip fracture, where the GR rose modestly with increasing age (*p*‐interaction = 0.15), and for mortality, where the effect size decreased with greater age (*p*‐interaction = 0.035). These associations are summarized in Table 4. There was no evidence for any interaction between follow‐up time and ALM/height^2^ for any of the fracture outcomes, incident falls, or mortality, ie, there was no evidence that the predictive effect of ALM/height^2^ was different at the beginning, compared with at the end, of the follow‐up period.

**Table 4:** Associations between DXA ALM/height^2^ and Incident Outcomes at Specific Ages

Age (years)	Ost fx	Hip fx	Falls	Death
All	**0.90 (0.85, 0.96)**	**0.81 (0.71, 0.91)**	0.98 (0.96, 1.01)	1.13 (1.08, 1.18)
50	0.91 (0.77, 1.08)	**0.60 (0.39, 0.91)**	1.03 (0.97, 1.08)	**1.32 (1.13, 1.54)**
60	0.91 (0.82, 1.00)	**0.67 (0.50, 0.88)**	1.00 (0.97, 1.03)	**1.25 (1.12, 1.38)**
70	**0.90 (0.84, 0.96)**	**0.75 (0.64, 0.87)**	**0.97 (0.95, 0.99)**	**1.18 (1.10, 1.25)**
80	**0.90 (0.81, 0.99)**	**0.84 (0.74, 0.95)**	**0.95 (0.91, 0.99)**	**1.11 (1.05, 1.17)**
*p*‐value interaction ALM/height^2^ x age on outcome	>0.30	0.15	0.092	**0.035**

DXA = dual‐energy X‐ray absorptiometry; ALM = appendicular lean mass; Ost = osteoporotic; fx = fracture.

Models are adjusted for age and follow‐up time alone. Associations where *p* < 0.05 are in bold. Data are gradient of risk (GR; hazard ratio per SD) and 95% confidence interval. Note that GR is calculated at each specific age from hazard functions.

## Discussion

Consistent with our findings in older men, we have demonstrated that greater DXA ALM/height^2^ is modestly predictive of lower risk of incident fractures but that this association is markedly attenuated by adjustment for femoral neck BMD. Indeed, there was evidence of inversion of the relationship after BMD *T*‐score adjustment such that greater ALM/height^2^ was associated with greater fracture risk. Interestingly, there was no association with incident falls. However, ALM/height^2^ was associated positively with risk of death during follow‐up in all models.

Our finding that lower DXA ALM/height^2^ was modestly predictive of greater fracture incidence independently of past falls and FRAX probability appears consistent with our recent observations among men in MrOS^(^ ^6^ ^)^ and with previous findings from the Health ABC study^(^ ^7^ ^)^ and Framingham study.^(^ ^9^ ^)^ In all three settings, the ALM‐fracture relationship was markedly attenuated by the addition of femoral neck BMD *T*‐score, whereas greater ALM (or ALM/height^2^) appeared to be a risk factor for hip fracture after accounting for femoral neck BMD. In contrast, in the Swiss GERICO study, adjustment of low lean mass for BMD did not substantially attenuate associations with incident fracture.^(^ ^16^ ^)^ In an earlier WHI analysis, participants were classified into mutually exclusive groups based on BMD and sarcopenia (dichotomous variable according to appendicular lean mass adjusted for height and fat mass) status.^(^ ^8^, ^17^ ^)^ Low BMD was associated with increased risk of hip fracture, but women with sarcopenia alone had similar hazard ratios for hip fracture to non‐sarcopenic women with normal BMD, suggesting that sarcopenia alone is not predictive of hip fracture. In a further WHI study of 872 participants 65 years or older who met Fried's criteria for frailty, appendicular lean mass was associated with incident hip fracture, but this association did not remain statistically significant after adjusting for hip BMD.^(^ ^18^ ^)^

There are several potential reasons why DXA appendicular lean mass might not have optimal predictive capacity for incident fracture outcomes.^(^ ^1^ ^)^ ALM represents all the tissue that is neither fat nor bone and thus includes contributions from non‐muscle connective tissue, skin, and ligaments.^(^ ^19^ ^)^ The correlation between ALM/height^2^ and femoral neck BMD in the present population was 0.41. This is similar to that we observed in men in the MrOS cohorts^(^ ^6^ ^)^ and consistent with our understanding of the underlying DXA algorithms and biology. Importantly, ALM and femoral neck BMD are derived from the same instrument, ie, DXA, and the nature of the algorithms used effectively means that a mathematical relationship between lean mass and BMD is inevitable.^(^ ^19^ ^)^ Finally, it is likely that those with lower lean mass also have lower bone mass, and there is a well‐established biomechanical relationship between muscle and bone, as described by the mechanostat hypothesis.^(^ ^20^ ^)^ This suggests positive causal adaptations of bone mass to muscle strain, and indeed, in the MrOS cohort, we observed that measures of physical performance such as gait speed and chair stand time, together with grip strength, appeared to be rather more robust predictors of incident fracture than did DXA ALM.^(^ ^6^ ^)^ Other studies have similarly demonstrated the greater predictive capacity of physical function over this estimate of muscle mass.^(^ ^21^, ^22^, ^23^, ^24^, ^25^ ^)^ Importantly, muscle quality and adiposity cannot be adequately assessed using DXA, and taken as a whole, these findings suggest that other measures of muscle, such as creatine dilution^(^ ^26^ ^)^ or muscle cross‐sectional area or density from (p)QCT,^(^ ^27^, ^28^ ^)^ might be usefully evaluated as measures of muscle mass.

Previous studies examining associations between baseline sarcopenia and incident falls have demonstrated either increased falls risk^(^ ^29^ ^)^ or no association with this exposure.^(^ ^30^, ^31^ ^)^ Indeed in a study of the predictive value of four sarcopenia definitions for falls‐related hospitalization in older Australian women, there was no association for sarcopenia definitions overall. In contrast the component measures of muscle strength and physical function, but not DXA ALM, were associated with falls risk.^(^ ^31^ ^)^ Few previous investigations have focused on appendicular lean mass. However, our finding of a lack of association between ALM/height^2^ and incident falls is consistent with recent results from MrOS, in which, whereas measures of skeletal muscle using creatine dilution were associated with injurious falls, there was little evidence of any predictive value for DXA ALM.^(^ ^26^ ^)^

Our finding of a positive association between ALM/height^2^ and incident mortality is possibly somewhat counterintuitive and contrasts with results from the Tasmanian Older Adult Cohort. Here, among 1041 women, mean age 63 years, low ALM/height^2^ was not associated with increased mortality, although the point estimate was in the opposite direction (low ALM, greater mortality) to that which we observed in our present analysis. Furthermore, low ALM divided by BMI was statistically significantly associated with greater mortality.^(^ ^30^ ^)^ In contrast, in our analysis, use of ALM/BMI yielded no evidence of association (data not shown). Our finding is consistent with associations demonstrated previously in WHI, between greater percentage lean body mass (ie, not absolute ALM) and increased mortality. However, this association was only observed in women aged 70 to 79 years, whereas the opposite association of lower risk of death with greater percentage lean body mass was observed in women 50 to 59 areas old.^(^ ^32^ ^)^ Conversely, in our analysis, we observed a tendency for the adverse relationship between ALM and mortality to be of greater magnitude at younger ages. Our greater ALM–greater mortality finding was consistent regardless of incorporation of ALM alone or ALM/height^2^ in the models, and robust to adjustment for fat mass or waist‐hip ratio. However, it is not altogether possible to fully account for an effect of fat mass, given the collinearity between fat and lean in both DXA measurement and biological terms; while more detailed understanding of these findings awaits investigation in other cohorts, these findings indicate the complexity of relationships between bone health, body composition, and functional measures as predictors of future outcomes.

We studied a very large, uniformly characterized cohort of older women. The exposure and outcomes were validated, and we were able to account for other potential confounding factors. However, there are some limitations that should be considered in the interpretation of our findings. First, the cohort consisted solely of older women, limiting generalizability. However, we undertook this analysis to evaluate whether our previous findings in older men also applied to older women. Second, we were not able to investigate other measures of muscle mass to evaluate their performance characteristics in comparison with DXA ALM lean mass. Third, secondary causes of osteoporosis were rare in this population and were difficult to align with FRAX definitions, and so this specific input variable was set to zero in the FRAX models. This is likely to have slightly reduced the overall FRAX probabilities but is unlikely to have influenced the relationships observed. Indeed, selection of secondary causes as an input variable in the FRAX model does not contribute to the output fracture probability when BMD is included in the FRAX calculation, since the core assumption is that these conditions contribute via BMD.^(^ ^33^ ^)^ Fourth, WHI has a complex structure comprising an observational cohort and trials of calcium and vitamin D versus placebo and hormone therapy versus placebo. Although we adjusted for randomization, it remains possible that there might be some residual effect of the interventions. Fifth, different DXA instruments were used in different centers over time, and it was not possible to scan participants above the manufacturer's weight limit for the instrument. Cross‐calibration was undertaken, but it is possible that these considerations might have attenuated the magnitude of associations we observed. Finally, we had limited ability to delineate underlying mechanisms in this study design. However, our primary objective was to elucidate risk relationships that would be useful in risk assessment. In this context, causality and mechanism are second‐order considerations, that is, for a risk factor to be useful in risk prediction, it is sufficient that it be associated with the outcome, regardless of whether the relationship is causal. Thus, although we did undertake post hoc exploratory analyses in an attempt to shed further light on the unexpected positive association between ALM/height^2^ and mortality, we did not therefore undertake such mechanistic analyses more widely.

In conclusion, we have confirmed in older women our recent finding in older men, that DXA appendicular lean mass is only modestly predictive of incident fracture outcomes and does not add fracture risk information over and above femoral neck BMD and FRAX probability. In contrast, greater ALM/height^2^ was associated with a modestly greater hazard of death during the follow‐up. Our findings suggest that the inclusion of DXA ALM in sarcopenia definitions contributes minimal predictive information for falls and fracture, supporting the approach taken in the most recent US^(^ ^4^, ^5^ ^)^ and European^(^ ^3^ ^)^ sarcopenia definitions.

## Disclosures

All authors state that they have no conflicts of interest.

### PEER REVIEW

The peer review history for this article is available at https://publons.com/publon/10.1002/jbmr.4239.

## Supporting information


**Table S1.** Baseline Characteristics by Quarter of ALM/height^2^
