# Supplementary material for: Predictive Value of DXA Appendicular Lean Mass for Incident Fractures, Falls, and Mortality, Independent of Prior Falls, FRAX, and BMD: Findings from the Women's Health Initiative (WHI)
Source: J Bone Miner Res. 2021 Jan 28;36(4):654–61. doi: 10.1002/jbmr.4239 (PMC7610603; doi:10.1002/jbmr.4239)
Supplement: Supplementary file 1 — Table S1. Baseline Characteristics by Quarter of ALM/height2 [file JBMR-36-654-s001.docx]

**Supplementary Table 1:** Baseline characteristics by quarter of ALM/height^2^

|  |  |  | **Quarters of ALM/height^2^** | | | |
| --- | --- | --- | --- | --- | --- | --- |
|  |  | **All**  **n=11187** | **1st quarter**  **n=2787** | **2nd quarter**  **n= 2796** | **3rd quarter**  **n=2824** | **4th quarter**  **n=2780** |
|  |  | **Mean (SD) or n (%)** | **Mean (SD) or n (%)** | **Mean (SD) or n (%)** | **Mean (SD) or n (%)** | **Mean (SD) or n (%)** |
| Age (years) | 11187 | 63.3 (7.4) | 64.1 (7.3) | 63.9 (7.6) | 63.4 (7.4) | 61.8 (7.1) |
| Height (cm) | 11187 | 161.6 (6.4) | 161.7 (6.2) | 161.6 (6.1) | 161.6 (6.2) | 161.5 (6.8) |
| BMI (kg/m^2)^ | 11180 | 28.2 (5.9) | 23.8 (3.4) | 26.0 (3.5) | 28.6 (4.1) | 34.5 (5.7) |
| Prior fracture | 7685 | 1325 (17%) | 380 (19%) | 358 (19%) | 326 (17%) | 261 (14%) |
| Parental history hip fracture | 10927 | 1326 (12%) | 370 (14%) | 372 (14%) | 323 (12%) | 261 (10%) |
| Current smoking | 11029 | 889 (8%) | 238 (9%) | 227 (8%) | 238 (9%) | 186 (7%) |
| Corticosteroids | 11187 | 98 (1%) | 30 (1%) | 29 (1%) | 23 (1%) | 16 (1%) |
| Rheumatoid arthritis | 10384 | 607 (6%) | 140 (5%) | 153 (6%) | 142 (5%) | 172 (7%) |
| Excess alcohol intake | 11151 | 324 (3%) | 98 (4%) | 85 (3%) | 96 (3%) | 45 (2%) |
| Femoral neck BMD (g/cm^2^) | 11187 | 0.72 (0.13) | 0.67 (0.11) | 0.70 (0.11) | 0.72 (0.12) | 0.80 (0.13) |
| FRAX MOF wo | 11187 | 9.8 (6.9) | 12.1 (7.6) | 11.0 (7.0) | 9.5 (6.3) | 6.7 (5.0) |
| FRAX MOF w | 11186 | 10.4 (7.7) | 12.6 (8.3) | 11.7 (8.2) | 10.3 (7.5) | 7.1 (5.5) |
| Prior falls | 10067 | 3307 (33%) | 829 (33%) | 811 (33%) | 842 (33%) | 825 (33%) |
| ALM (g) | 11187 | 14769 (2809) | 11909 (1204) | 13679 (1121) | 15229 (1293) | 18264 (2349) |
| ALM/height^2^ (g/cm^2^) | 11187 | 0.56 (0.10) | 0.45 (0.03) | 0.52 (0.02) | 0.58 (0.02) | 0.70 (0.08) |
| **During follow-up** |  |  |  |  |  |  |
| Length of follow-up (years) | 11187 | 14.2 (5.5) | 14.2 (5.5) | 14.5 (5.5) | 14.3 (5.5) | 13.9 (5.6) |
| Any fracture | 11187 | 1692 (15%) | 460 (17%) | 444 (16%) | 432 (15%) | 356 (13%) |
| Osteoporotic fracture | 11187 | 1225 (11%) | 343 (12%) | 328 (12%) | 318 (11%) | 236 (8%) |
| MOF | 11187 | 1024 (9%) | 292 (10%) | 282 (10%) | 266 (9%) | 184 (7%) |
| Hip fracture | 11187 | 344 (3%) | 112 (4%) | 99 (4%) | 87 (3%) | 46 (2%) |
| Falls | 11144 | 7720 (69%) | 1965 (71%) | 1928 (69%) | 1929 (69%) | 1898 (69%) |
| Death | 11187 | 2236 (20%) | 552 (20%) | 539 (19%) | 560 (20%) | 585 (21%) |

MOF=Major Osteoporotic Fracture
